# Notes and Notices

**Published:** 1887-10

**Authors:** 


### NOTES AND NOTICES.

Removal.—Dr. Samuel A. Kimball, of Boston, has removed his offices from Boylston Street to 124 Commonwealth Avenue.

Medicines *vs.* Doctors.—It is true that doctors disagree, but they do not disagree half so much as do their medicines!—*Free Press.*

Uses of the Tongue.—Doctors examine the tongue to find out the diseases of the body, philosophers to find out the diseases of the mind!

The Southern Homœopathic Medical Association will hold its fourth annual meeting in New Orleans December 14th–16th, 1887.

C. G. Fellows, M. D., *Secretary,*
New Orleans, La.

Correction.—July number, page 260, line 15 from top, should read thus: Dr. Schmitt remarked that he gave Pulsatilla when the woman cries, "Oh! my back! Oh! my back!" like Causticum, but there is not the exhaustion from previous sickness as under Causticum. She wants her back pressed like Kali-carb., but the pains go from back to front, not down the buttocks, as under Kali-carb.

Infection from Dairy Products.—In the *Scientific American* of July 16th, 1887, is an article under the above heading, detailing the investigations of V. Gaulthier, a French chemist. It was found that such articles of diet from cows affected with tubercular disease communicated phthisis or consumption to poultry and swine, and "could become thus directly or indirectly a serious menace to man."

"The result of some of the more recent observations is that cows may themselves become infected with a sickness resembling scarlet fever, and that such cows may, by their milk, cause the true scarlet fever to be developed in human beings.

"In one instance an outbreak of scarlet fever was associated with a certain dairy. * * * The disease was attributed to certain cows. Examination of them showed the presence of disease, whose symptoms included sores upon the body, ulcerations, and a visceral complaint resembling that occurring in scarlet fever in the human being. * * * Their disease, so similar to human scarlet fever, made it almost certain that they were the origin of the trouble."

The Homœopathic Medical Department of the State University of Iowa has just issued a circular, from which we extract the following: "At a recent meeting of the Board of Regents a full Chair of Surgery was established in the Homœopathic Department of the State University, to sustain which it is imperatively necessary to provide a hospital for the care of the patients that may apply for clinical treatment. Accordingly, a State Hospital Association has been incorporated membership in which is to be secured by the payment of five dollars annually, giving the right to vote on all matters pertaining to the management of the hospital, and to hold office in the Association. Patrons, with the privilege of a free bed at their command, can be enrolled on the list of members by the payment of three hundred dollars a year, which sum, it is estimated, will cover the cost of board, nursing, and treatment for one patient one year. Life members can secure all of the above privileges for their life-time by the payment of one thousand dollars in one payment."

Difficult Respiration.—On page 378 of this number Dr. Ballard mentions the symptom of Pulsatilla: "Difficult respiration accompanies diseased conditions in parts not involved in the act of breathing." We can confirm this symptom in two or three cases in our own practice. The most prominent one was that of a young girl with menstrual colic. The above indication, appearing amid a number of symptoms of doubtful value in selecting the remedy, cleared up the case at once. We gave Puls. and brought about immediate relief. W. M. J.

Kent's Lectures.—We are glad to note that Professor Kent's lectures are being translated and published by both the *Rivista Omiopatica*, of Rome, and *L'Union Medicale*, of Antwerp. As these lectures are much appreciated in this country, we hope they will be found as interesting and serviceable to our foreign brethren.

Dr. Higgins' Clinical Note.—This instructive note, which will be found on page 390, was sent to us last January. By an oversight it has remained in our safe for the past seven months.

International Medical Congress.—For the first time in its history the International Medical Congress of the old school met in America. Its sessions were opened by the President and an address was given by the Secretary of State. Mr. Bayard, being an homœopathist, should have told these learned physicians how to *really* cure patients, but all the same he did not!

Opprobrium Medicorum.—This title has usually been given to surgery, but the *Pacific Record* appropriately applies it to gonorrhœa, referring, of course, to the allopathic maltreatment of it. Correct for once!

The Human Family.—The New York *Commercial News* says: "The human family living to-day on earth consists of about one billion four hundred and fifty million individuals; not less, probably more. These are distributed over the earth's surface, so that now there is no considerable part where man is not found." Only those of the lowest grades of civilization are not subscribing to The Homœopathic Physician!

